# Combined effects of exercise and different levels of acute hypoxic severity: A randomized crossover study on glucose regulation in adults with overweight

**DOI:** 10.3389/fphys.2023.1174926

**Published:** 2023-04-13

**Authors:** Chris Chow Li Tee, Evelyn B. Parr, Matthew B. Cooke, Mee Chee Chong, Nurhamizah Rahmat, Mohd Rizal Md Razali, Wee Kian Yeo, Donny M. Camera

**Affiliations:** ^1^ Division of Research and Innovation, National Sports Institute of Malaysia, Kuala Lumpur, Malaysia; ^2^ Sport and Exercise Medicine Group, Swinburne University of Technology Melbourne, Hawthorn, VI, Australia; ^3^ Exercise and Nutrition Research Program, Mary Mackillop Institute for Health Research, Australia Catholic University, Melbourne, VI, Australia

**Keywords:** hypoxia, low-intensity exercise, exerkines, apelin, FGF-21

## Abstract

**Purpose:** The aim of this study was to investigate the influence of manipulating hypoxic severity with low-intensity exercise on glucose regulation in healthy overweight adults.

**Methods:** In a randomized crossover design, 14 males with overweight (age: 27 ± 5 years; body mass index (BMI) 27.1 ± 1.8 kg⋅m^2^) completed three exercise trials involving 60 min aerobic exercise cycling at 90% lactate threshold in normoxia (NM, FiO_2_ = 20.9%), moderate hypoxia (MH, FiO_2_ = 16.5%) and high hypoxia (HH, FiO_2_ = 14.8%). A post-exercise oral glucose tolerance test (OGTT) was performed. Venous blood samples were analyzed for incremental area under the curve (iAUC), plasma glucose and insulin, as well as exerkine concentrations (plasma apelin and fibroblast growth factor 21 [FGF-21]) pre- and post-exercise. A 24-h continuous glucose monitoring (CGM) was used to determine interstitial glucose concentrations. Heart rate, oxygen saturation (SpO_2_) and perceptual measures were recorded during exercise.

**Results:** Post-exercise OGTT iAUC for plasma glucose and insulin concentrations were lower in MH vs. control (*p* = 0.02). Post-exercise interstitial glucose iAUC, plasma apelin and FGF-21 were not different between conditions. Heart rate was higher in HH vs. NM and MH, and MH vs. NM (*p* < 0.001), while SpO_2_ was lower in HH vs. NM and MH, and MH vs. NM (*p* < 0.001). Overall perceived discomfort and leg discomfort were higher in HH vs. NM and MH (*p* < 0.05), while perceived breathing difficulty was higher in HH vs. NM only (*p* = 0.003).

**Conclusion:** Compared to higher hypoxic conditions, performing acute aerobic-based exercise under moderate hypoxia provided a more effective stimulus for improving post-exercise glucose regulation while concomitantly preventing excessive physiological and perceptual stress in healthy overweight adults.

## Introduction

The current obesity epidemic is a global health issue evidenced through a tripling of obesity incidence since 1975 and has emerged as the leading cause of non-communicable diseases (WHO, 2021). Individuals with overweight or obesity, defined as body mass index (BMI) ≥25 and ≥30 kg⋅m^2^, respectively, are at higher risk of impaired metabolic homeostasis, reduced insulin sensitivity (Krogh-Madsen et al., 2010) and postprandial lipid metabolism (Booth et al., 2012), which can be contributed by physical inactivity (WHO, 2021). Regular physical activity or exercise exerts numerous health benefits such as improved cardiovascular fitness, anabolic (e.g., increased muscle mass) (Callahan et al., 2021) and metabolic (e.g., enhanced mitochondrial biogenesis and substrate metabolism) adaptations (Coffey and Hawley, 2007; Gibala and McGee, 2008; MacInnis and Gibala, 2017), and reduced levels of circulating pro-inflammatory markers that collectively reduce all-cause mortality and improve quality of life (Chakravarty et al., 2008; Hawley et al., 2014).

Hypoxic exposure (i.e., reduction of oxygen supply to tissues) combined with exercise can synergistically increase adaptations associated with exercise (Tee et al., 2023). For instance, elite athletes regularly engage in hypoxic training to improve muscular adaptations (i.e., citrate synthase, mitochondria density) (Hoppeler et al., 2008), physiological regulatory systems (i.e., haemoglobin mass) and ultimately, physical performance (Wilber, 2007; Saunders et al., 2009). In clinical settings, acute (i.e., single bout) (Mackenzie et al., 2011; Mackenzie et al., 2012b) and chronic (i.e., 3 times/week for 4–6 weeks) (Haufe et al., 2008; Wiesner et al., 2010; De Groote et al., 2018) exercise in combination with hypoxia (∼2,700–3,100 m) can lead to improved metabolic health markers (e.g., plasma glucose and insulin), body compositional changes and cardiorespiratory health in people with overweight or obesity (Fernandez Menendez et al., 2018). These beneficial effects are of greater relevance to people with overweight or obesity considering the addition of hypoxia to exercise training can induce similar or greater physiological adaptations despite a lower absolute exercise intensity (Fernandez Menendez et al., 2018). Moreover, greater metabolic stress induced at a lower exercise intensity under hypoxic conditions can also reduce muscle/joint load and mechanical strain, which is important considering such extraneous strain is an established source of injury and exercise reluctance due to increased body mass in cohorts with overweight and obesity (Millet et al., 2016; Pramsohler et al., 2017; Jung et al., 2021; Tee et al., 2022).

While the underlying mechanisms of blood glucose regulation with combined exercise and hypoxia remain largely unknown, exercise-secreted factors (i.e., “exerkines”) may play a role in orchestrating this process (Chow et al., 2022). Exerkines such as apelin and fibroblast growth factor 21 (FGF-21) have been implicated in mediating improvements in insulin sensitivity and substrate metabolism with exercise, respectively (Carson, 2017; Garneau and Aguer, 2019; Laurens et al., 2020). Studies have reported that acute aerobic exercise under normoxia can regulate the secretion of apelin (Besse-Patin et al., 2014; Son et al., 2019) and FGF-21 (Kim et al., 2013; Taniguchi et al., 2016; Tanimura et al., 2016) in humans. While the effects of exercise under hypoxia on circulating apelin and FGF-21 remain unknown, hypoxia stimulation in cultured adipocytes has been shown to induce the secretion of apelin (Glassford et al., 2007; Geiger et al., 2011) and FGF-21 (Wu et al., 2020). Such data suggest that the secretion of exerkines such as apelin and FGF-21 could be modulated by combined exercise and hypoxia. To the best of our knowledge, no studies have examined the plasma concentrations of apelin and FGF-21 in response to combined exercise and hypoxia. Therefore, it is important to examine the changes in these exerkines to understand the potential mechanisms of blood glucose regulation in response to combined exercise and hypoxia.

Accumulating research demonstrates numerous health benefits from performing acute or chronic exercise under low to moderate hypoxic conditions. However, some evidence suggests that exercising at severe levels of hypoxia (>3,000 m) may lead to detrimental physiological responses such as increased oxidative stress and inflammation (Ristow and Schmeisser, 2014; Burtscher et al., 2022). While combined exercise and hypoxia can improve blood glucose regulation (Mackenzie et al., 2011; De Groote et al., 2018), there is no current consensus in the literature regarding the effective level of hypoxia to induce the greatest improvements. Previous studies have shown either decreased (Lei et al., 2022) or increased blood glucose levels (Kon et al., 2015) following sprint interval exercise in healthy active participants, regardless of different hypoxic levels. However, no study to date has compared different levels of hypoxic stimuli combined with acute low-intensity exercise on glucose tolerance, or incorporated continuous blood glucose monitoring, to provide a more comprehensive evaluation of whether potential improvements in blood glucose responses can be optimized with different levels of hypoxic severity in overweight adults (Tee et al., 2023). Therefore, the primary aim of this study was to investigate the acute effect of normoxia, moderate hypoxia, or high hypoxia combined with low-intensity aerobic cycle exercise, on blood glucose regulation. A secondary aim of our study was to determine whether different levels of hypoxic stimuli differentially regulate the expression of apelin and FGF-21. Compared to normoxia and high hypoxia, we hypothesized that the addition of a moderate hypoxic stimulus to low-intensity exercise would improve glucose regulation and exerkines in adults with overweight.

## Methods

### Participants

Fourteen overweight, physically inactive males (mean ± SD, age: 27 ± 5 years; height 175 ± 0 cm; body mass (BM) 83.0 ± 7.0 kg; body mass index (BMI) 27.1 ± 1.8 kg⋅m^2^; body fat percentage (BF) 28.4% ± 2.5%; lactate threshold (LT) 90 ± 25 W), participated in this study after meeting the eligibility criteria. Eligible participants were those with a BMI between 25.0–29.9 kg⋅m^2^, normotensive (90–120 and 60–80 mmHg systolic and diastolic blood pressure, respectively), no known cardiovascular, metabolic, or physiological disease, physically inactive (<150 min/week of physical activity), and no exposure to altitude (≥1,000 m) within 3 months prior to participation. Participants were screened for eligibility and their physical activity levels were measured using the Adult Pre-exercise Screening System (APSS) (Exercise & Sports Science Australia, 2019). This study was approved by the Human Research Ethics Committee of the National Sports Institute of Malaysia (ISNRE/A/008/2020–003/2020), Swinburne University of Technology (Australia) (20225950-9155) and registered on ClinicalTrials.gov (trial identifier: NCT05577429). The study was undertaken in accordance with the *Declaration of Helsinki* and written informed consent was obtained from each participant.

### Experimental protocol

**FIGURE 1:**
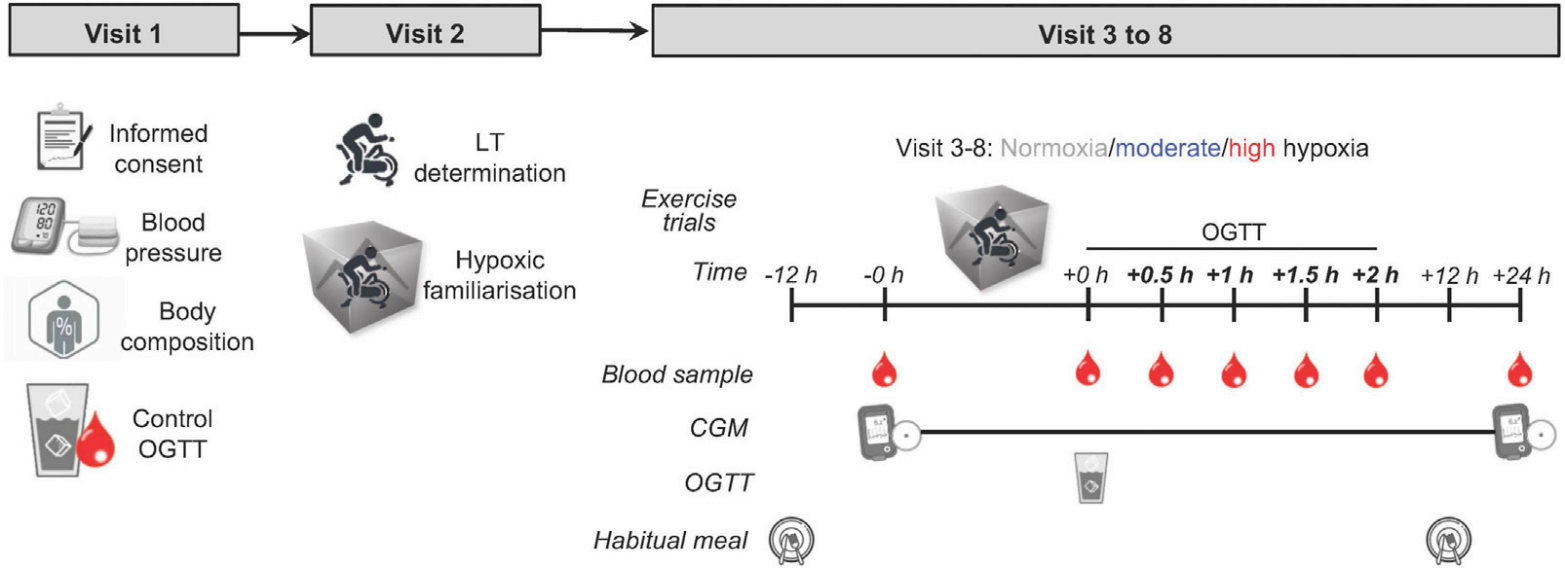
Study protocol: an overview of the entire study. Eligible healthy, overweight participants (*n* = 14) completed three exercise trials in three altitude conditions in a randomized order separated by ≥ 7 days 75 g of glucose dissolved in water was provided immediately following each exercise trial for the measurement of OGTT. Blood samples were collected pre, immediately post, every 0.5 h for 2 h post and post-24 h each exercise trial. OGTT, oral glucose tolerance test; LT, lactate threshold test; CGM, continuous glucose monitor.

An overview of the experimental protocol is shown in Figure 1. Participants attended the laboratory for a total of eight visits (two baseline visits, three exercise trials, and three post-24 h follow-up visits). Throughout the experimental period, participants were instructed to maintain their habitual diet and activities of daily living. For each visit, participants were instructed to attend the laboratory following an overnight fast for at least 10 h. During the first visit, blood pressure and body composition were measured and a control (CTL) 2 h oral glucose tolerance test (OGTT) was conducted (described subsequently). Body composition parameters including body mass, height, and body fat percentage (BF) were measured using bioimpedance analysis (Inbody 770, Cerritos, CA, United States) in light clothing.

During the second visit, participants were asked to perform a lactate threshold (LT) test. Participants cycled on a cycle ergometer (Velotron Racermate, Seattle, United States) with an initial load of 50 W. The load was increased progressively in 15 W increments every 4 min, with participants maintaining a constant pedal frequency (cadence ∼90 rpm) until reaching LT, which is defined as the power output preceding a sudden and sustained increase in lactate (≥1.0 mmol/L greater than baseline), as described previously (Farrell et al., 1979). Heart rate (HR) was recorded throughout the test using a heart rate monitor (Polar H10, Polar Electro OY, Kempele, Finland). Ratings of perceived exertion (RPE) were recorded at the end of every 4 min stage using Borg 1–10 scale. The first and second visits were conducted at least a week prior to the third visit (exercise trial).

For visits 3 to 8, three exercise trials were carried out in a single-blinded randomized crossover design. The order of conditions was determined by random assignment and implemented by an investigator who was not involved with the exercise trials. Each trial was separated by at least 7 days. All exercise trials were conducted at 7:30 a.m. The exercise trials consisted of a 60-min cycling bout at 90% of LT under three simulated hypoxic conditions: i) normoxia near sea level (NM; FiO_2_ = 20.9%), ii) normobaric moderate hypoxia (MH; FiO_2_ = 16.5% corresponding to a simulated altitude of ∼2000 m), and iii) normobaric high hypoxia (HH; FiO_2_ = 14.8%, ∼3,000 m). Considering excessive hypoxia (severe, >3,000 m) can cause adverse health effects such as high-altitude sickness, the levels of hypoxia selected in our study were chosen based on safety and practical reasons while also ensuring adequate metabolic stress was induced (Millet et al., 2016; Burtscher et al., 2022). All exercise trials were conducted in an environmental chamber (Welltech Instruments, Hong Kong) with temperature and relative humidity maintained at 20 °C and 40%, respectively.

### Measurements on exercise trial days

A continuous glucose monitor sensor (CGM; FreeStyle Libre™, Abbott Diabetes Care, Witney, UK) was placed on the back of the upper arm, according to the manufacturer’s instructions. Participants were instructed to scan the sensor with a CGM reader every 8 h to minimize missing data.

Venous blood samples (EDTA) were collected from antecubital vein via venipuncture at pre-, immediately post- (within 5 min after the cessation of exercise), and 24 h post-exercise. During the cycling exercise, HR and oxygen saturation (SpO_2_) were recorded. After the exercise, participants were asked to reflect on their subjective perceptions, including their ratings of overall perceived discomfort, perceived breathing difficulty, and leg discomfort, using modified Borg CR10 scales (Tee et al., 2022). Symptoms of acute mountain sickness were assessed using the Lake Louise Questionnaire (Roach et al., 2018) at the end of the exercise. Participants displayed no symptoms of acute mountain sickness during hypoxic exposures.

### Oral glucose tolerance test (OGTT)

A 2 h oral glucose tolerance test was performed immediately after exercise. A 20-gauge cannula was inserted into the dorsal hand vein for the collection of arterialized-venous blood. Participants consumed 75 g of glucose (Glucolin glucose powder) dissolved in 250 ml of water. Venous blood samples (EDTA and sodium fluoride) were drawn immediately post glucose consumption and at 30-min intervals up to 120 min (30, 60, 90, and 120 min).

### Biochemical analysis

Upon collection, blood samples were centrifuged at 2,000 x *g* for 10 min at 4 °C. Plasma glucose was measured using a biochemistry analyser (YSI 2900D Biochemistry Analyzer, Yellow Springs, OH, United States) with a coefficient of variation (CV) of <2.0%. Commercially available enzyme-linked immunoassay kits were used to analyse plasma insulin (Insulin ELISA, DE2935, Demeditec, Germany), plasma apelin (Apelin-12 EIA, EK-057–23, Phoenix Pharmaceuticals, Inc., CA, United States; *n* = 8), and plasma FGF-21 (Quantikine Human FGF-21 ELISA, DF2100, R&D Systems, MN, United States; *n* = 8) concentrations. The intra-assay CV was <2.6%, <10.0% and <3.9% for insulin, apelin and FGF-21, respectively, whereas the inter-assay variability was <6.0%, <15.0%, and <10.9%. All assays were performed according to the manufacturer’s instructions and were measured in duplicate.

### Statistical analysis

Sample size estimation was determined from *a priori* power analysis, using software G*Power (v3.1.9.7) to detect differences (effect size = 0.58, power of 0.80, alpha of 0.05) based on previous work comparing the effects of exercise cycling under normoxic and normobaric hypoxic (FiO_2_ = 14.6%) conditions on the glucose area under the curve in response to an intravenous glucose tolerance test (Mackenzie et al., 2011). It was determined that 12 participants were required, with 14 recruited allowing for a 20% attrition rate. Incremental area under the curve (iAUC) for 2 h OGTT venous plasma glucose and insulin concentrations were calculated using the trapezoid method (Potteiger et al., 2002). Total AUC (AUC_total_) for 24 h interstitial glucose derived from CGM was calculated using the trapezoid method. All data were analyzed using one-way repeated-measures analysis of variance (ANOVA) to compare between hypoxic conditions. Physiological and perceptual measures between hypoxic conditions were analyzed using one-way repeated-measures ANOVA. Differences over time (pre, post, and 24 h post) and conditions (NM, MH and HH) of plasma apelin and FGF-21 concentrations were evaluated by two-way repeated measures of ANOVA. Bonferroni-adjusted *p* values were performed if the main effect was observed. Effect sizes were described in terms of partial eta-squared (η^2^, with η^2^ ≥ 0.06 representing a moderate effect and η^2^ ≥ 0.14 a large effect) (Cohen, 2013). Due to our sample size (n < 20), Hedge’s *g* effect sizes were assessed to determine meaningful differences (with *g* = 0.38–0.75 representing a moderate effect and *g* ≥ 0.76 representing large effect sizes) (Brydges, 2019). All data are expressed as mean ± SD. All statistical tests were carried out using GraphPad Prism version 9.2.0 (GraphPad Software, San Diego, CA). Statistical significance was set at the level of *p* < 0.05.

## Results

### Participant characteristics and energy intake

Baseline characteristics of participants are presented in Table 1. Participants complied with recording 24-h dietary intake on exercise trial days, with no differences observed between conditions for macronutrients and energy intake (*p* > 0.05; Figure A and B, Supplementaary Figure S1).

**TABLE 1:** Participant characteristics.

Variables	Males (*n* = 14)
Fasting concentration	
Fasting glucose (mmol/L)	4.9 ± 0.4
Fasting insulin (µU/mL)	7.2 ± 1.9
HOMA-IR	1.6 ± 0.5
Systolic blood pressure (mm Hg)	116 ± 7
Diastolic blood pressure (mm Hg)	76 ± 5
Physical activity	
Time (min/week)	95 ± 59
Cycling lactate threshold power output W)	90 ± 25

Values are presented as mean ± SD.

HOMA-IR, homeostatic model assessment for insulin resistance.

### Biochemical analyses

**FIGURE 2:**
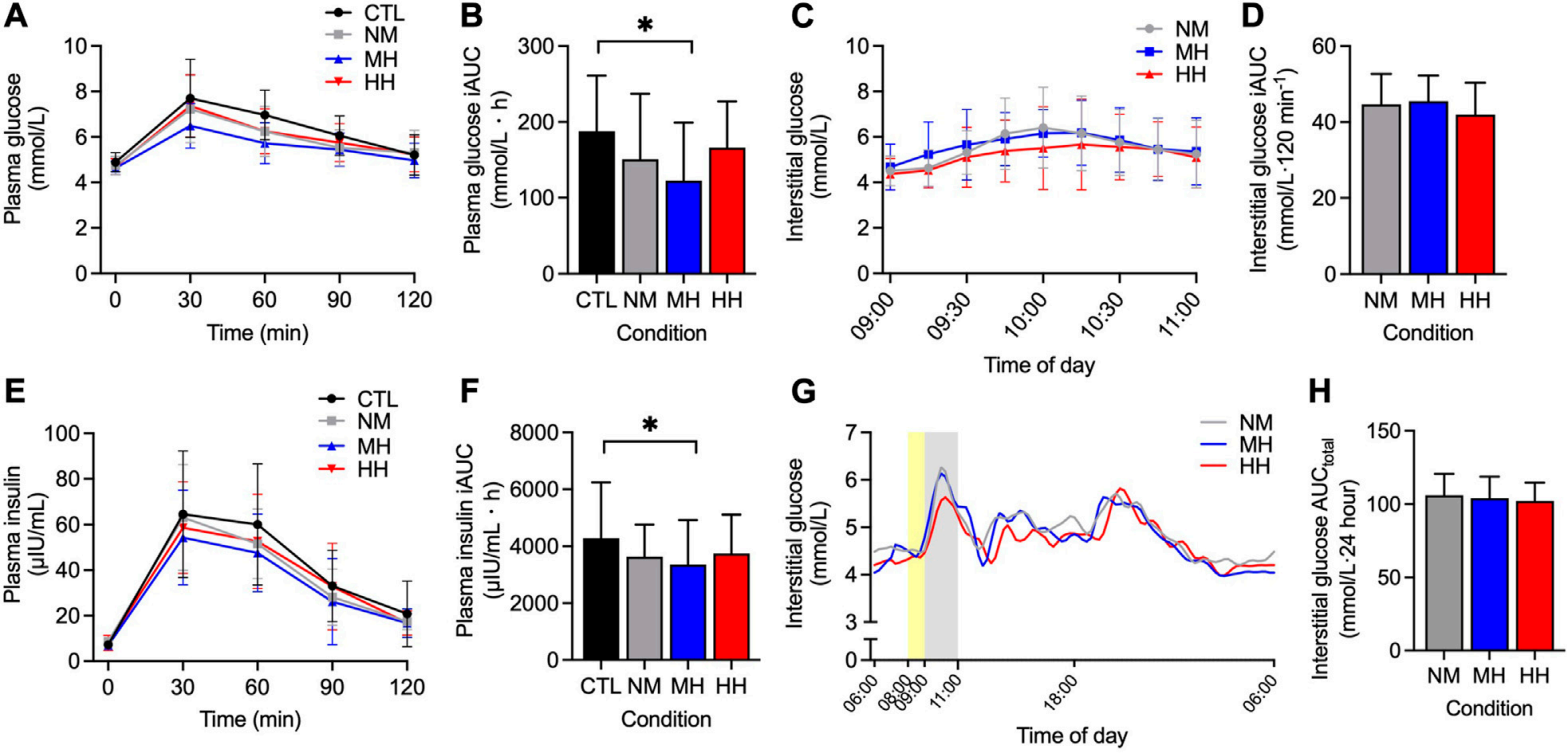
Two-hour responses following an OGTT after each of three exercise altitude conditions for venous plasma glucose **(A)**, interstitial (CGM) glucose **(C)**, plasma insulin **(E)** concentrations and the subsequent 2 h incremental AUC for plasma glucose **(B)**, plasma insulin **(F)** and interstitial glucose **(D)** concentration. 24 h interstitial (CGM) glucose concentration **(G)** and CGM AUC **(H)** from 06:00 until 06:00 the following morning. Values are mean ± SD. *p < 0.05 denotes a statistically significant difference between conditions. Area shaded with yellow represents 1 h exercise and grey represents OGTT. AUC, area under the curve; CGM, continuous glucose monitor; CTL, control; HH, high hypoxia; MH, moderate hypoxia; NM, normoxia; OGTT, oral glucose tolerance test.

There was a significant main effect of hypoxic condition on plasma glucose iAUC (*p* = 0.02; η^2^ = 0.28; Figure 2A). Plasma glucose iAUC was significantly lower in MH compared to CTL (−35% ± 4%; *p* = 0.046; *g* = 0.84; Figure 2B). No significant difference was observed in either NM or HH (−20% ± 18%; −12% ± 17%; *p* > 0.05, respectively) compared to CTL as well as between NM and MH or HH, or MH and HH (−19% ± 11%; +10 ± 30%; +35 ± 20%; *p* > 0.05, respectively). There was no difference between conditions for interstitial glucose iAUC during 2-h OGTT (Figure 2C), despite it was lower in HH compared to MH and NM (−8 ± 25%; −6 ± 6%; *p* > 0.05, respectively; Figure 2D). A main effect of hypoxic condition was detected for plasma insulin iAUC (*p* = 0.02; η^2^ = 0.24; Figure 2E). Plasma insulin iAUC was significantly lower in MH compared to CTL (−22% ± 20%; *p* = 0.03; *g* = 0.51; Figure 2F). No significant differences were observed between NM or HH and CTL (−15% ± 43%; −13% ± 30%; *p* > 0.05, respectively), as well as NM compared to either MH or HH, or between MH vs. HH (−8 ± 39%; +3 ± 22%; +11 ± 12%; *p* > 0.05, respectively). For interstitial glucose 24 h AUC_total_, no differences between conditions were detected (*p* > 0.05; Figures 2G, H).

**FIGURE 3:**
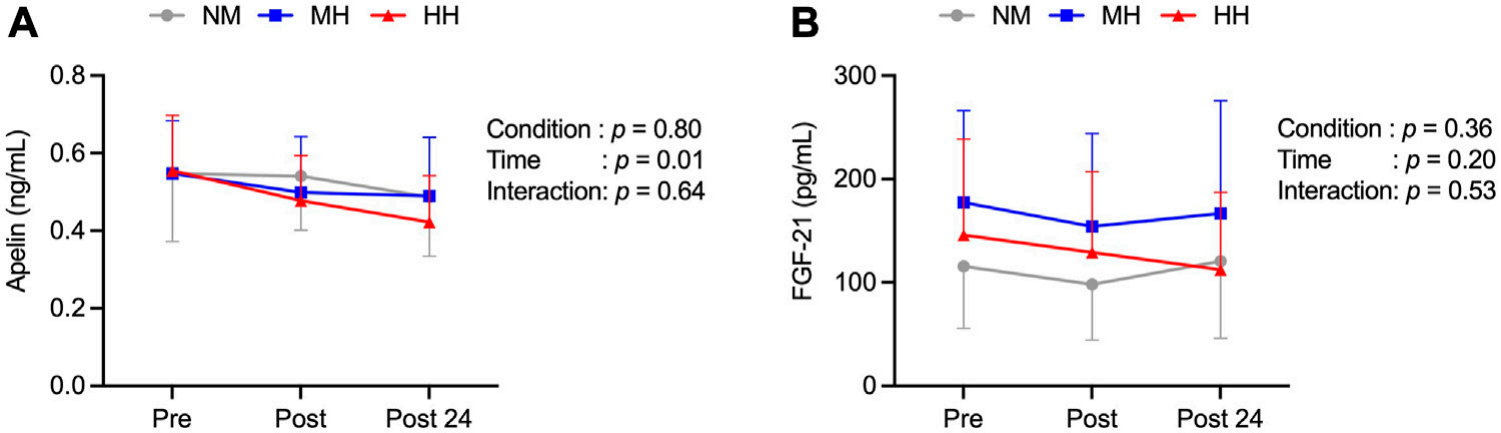
Pre, immediately post and 24 h post-exercise concentration of plasma apelin **(A)** and plasma FGF-21 **(B)** at three exercise altitude conditions (*n* = 8). Values are mean ± SD.

There was a main effect of time on plasma apelin (*p* = 0.01; Figure 3A), however, *post hoc* analysis did not reveal any significant changes between time points. No differences in hypoxic condition as well as time × hypoxic condition interaction effects were observed for plasma apelin (*p* > 0.05). Similarly, there were no differences in time, hypoxic condition as well as interaction effects for plasma FGF-21 (*p* > 0.05; Figure 3B).

### Physiological measures

**FIGURE 4:**
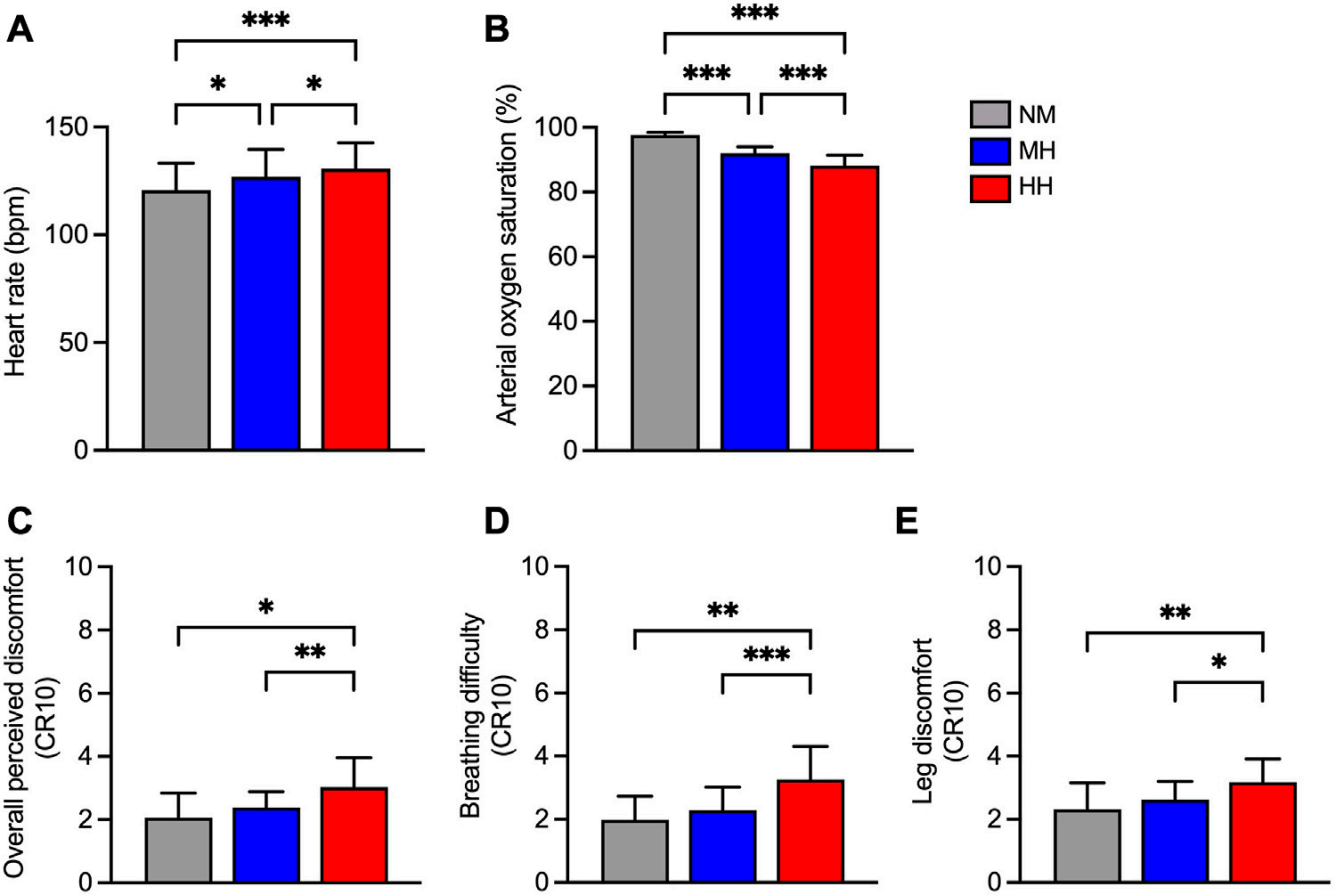
Heart rate **(A)**, arterial oxygen saturation (SpO_2;_ **(B)**, overall perceived discomfort **(C)**, difficulty breathing **(D)** and leg discomfort **(E)** at three exercise altitude conditions. Values are mean ± SD. *p < 0.05, **p < 0.01, ***p < 0.001 denotes a statistically significant difference between condition.

There were significant differences between hypoxic conditions for HR (*p* < 0.001; η^2^ = 0.61; Figure 4A), with higher HR responses in HH compared to both NM (8% ± 5%; *p* < 0.001; *g* = 0.78) and MH (3% ± 6%; *p* = 0.03; *g* = 0.29), as well as between MH and NM (*p* = 0.01; *g* = 0.47). There were also significant differences between hypoxic conditions for SpO_2_ (*p* < 0.001; η^2^ = 0.87; Figure 4B). SpO_2_ was significantly lower in HH (88.1% ± 3.3%) compared to both NM (97.1% ± 0.7%, *p* < 0.001; *g* = 3.89) and MH (92.1% ± 1.9%; *p* < 0.001; *g* = 1.41), as well as between MH and NM (*p* < 0.001; *g* = 3.74).

### Perceptual measures

There were significant differences between hypoxic conditions for overall perceived discomfort rating (*p* < 0.01; η^2^ = 0.41; Figure 4C). Overall perceived discomfort rating was greater in HH compared to both NM and MH (+32 ± 17%; *p* = 0.02; *g* = 1.09; +21 ± 45%; *p* = 0.005; *g* = 0.68, respectively). No significant differences were observed in overall perceived discomfort between MH and NM (+13 ± 51%; *p* > 0.05). Significant differences were found between hypoxic conditions for perceived breathing difficulty (*p* < 0.001; η^2^ = 0.55; Figure 4D) and perceived leg discomfort rating (*p* < 0.01; η^2^ = 0.42; Figure 4E). Perceived breathing difficulty and leg discomfort rating were greater in HH compared to both NM and MH (+39 ± 29%; *p* = 0.003; *g* = 1.37; +30 ± 30%; *p* < 0.001; *g* = 1.05; +27 ± 13%; *p* = 0.008; *g* = 1.07; +18 ± 20%; *p* = 0.03; *g* = 0.82, respectively). No significant difference was observed in either perceived breathing difficulty or leg discomfort rating in MH compared to NM (+17 ± 45%; +12 ± 42%; *p* > 0.05, respectively).

## Discussion

Our study has demonstrated that low-intensity cycle exercise performed under moderate hypoxia (2000 m) improved post-exercise OGTT plasma glucose and insulin iAUC compared to near sea-level and high hypoxia (3,000 m). Furthermore, HR and perceptual responses (i.e., overall perceived discomfort, perceived breathing difficulty and leg discomfort) were lower and SpO_2_ was higher with moderate hypoxia as compared to high hypoxia. Collectively, low-intensity exercise in combination with moderate hypoxia may provide effective conditions for enhancing acute blood glucose responses without inducing superfluous levels of respiratory and lower extremity discomfort in physically inactive and overweight adults.

Fasting glucose and insulin levels as well as insulin sensitivity have been previously demonstrated to be improved with low-intensity cycle exercise performed under high hypoxia (∼3,000 m) in individuals with type 2 diabetes (T2D) and overweight (Mackenzie et al., 2011; Mackenzie et al., 2012a). Our study is the first to compare different levels of hypoxic exposure as an adjuvant to exercise and we provide new information to indicate that significant improvements in acute blood glucose regulation can be attained with low-intensity cycle exercise without exposure to high or severe levels of hypoxia (i.e., >3,000 m). Such knowledge can be practically relevant for individuals with overweight or obesity. For instance, previous research has indicated that such population cohorts lose their enjoyment of exercise when exercise intensity is 10% greater than a self-selected speed (Ekkekakis and Lind, 2006). As adequate cardiorespiratory stimulation to induce metabolic adaptations can be achieved with lower exercise intensities when performed in combination with hypoxic loading (Hobbins et al., 2021; Tee et al., 2022), individuals with overweight or obesity may have better exercise adherence under such conditions compared to exercise with greater intensity undertaken in normoxia. Furthermore, in adults with overweight and T2D, acute hypoxic exercise was more effective than normoxic exercise at improving glucose tolerance (Mackenzie et al., 2011). While we acknowledge the current study is acute in nature, the findings provide an initial basis to show that overweight adults performing low-intensity cycle exercise at moderate hypoxia, as compared to normoxia or high hypoxia, can also promote significant improvements in post-exercise 2 h OGTT plasma glucose and insulin iAUC responses. Incorporating exercise and moderate hypoxia into habitual training program of adults with overweight and/or pre-diabetes may thus provide an effective approach to prevent potential later incidences of hyperglycemia and reduce their risk of developing diabetes.

In contrast to the observed venous plasma glucose responses, no differences between hypoxic conditions were apparent in the post-exercise interstitial glucose response to the OGTT using CGM. Disparities between venous and interstitial blood glucose measures have been reported previously (Cengiz and Tamborlane, 2009). Such differences may relate to the interstitial glucose reading from the CGM having a delayed effect due to glucose in the blood taking time to appear in the interstitial fluid (Rebrin et al., 2010), as well as the intervals of readings (15 min for CGM vs. 30 min for venous blood). When assessing 24 h CGM data, although not statistically significant, the AUC_total_ showed a trend to decrease after exercising in both hypoxic conditions as compared to normoxia. Of note, the 24-h macronutrient and energy intake during the exercise day were not significantly different between conditions, eliminating the confounding effects of dietary intake on blood glucose regulation. Together, our data suggest that adding hypoxia to exercise may help improve blood glucose regulation. To the best of our knowledge, this is the first investigation of 24 h blood glucose patterns in response to a combined exercise and hypoxia stimulus incorporating CGM measures. Whether further beneficial effects in daily blood glucose responses manifest with repeated exercise sessions performed under moderate hypoxia remains an area of future investigation.

Our current study also observed changes in physiological (e.g., heart rate and arterial oxygen saturation) and perceptual responses that have important implications for exercise adherence. As expected, HR significantly increased during low-intensity exercise under acute exposure to moderate and high hypoxia. Similarly, SpO_2_ values were progressively lower as hypoxic severity increased. Perceptual responses (i.e., ratings of overall perceived discomfort, perceived breathing difficulty and leg discomfort) were greater as hypoxic severity increased. These results are in line with previous observations examining physiological and perceptual responses to interval walking in adults with overweight and obesity at high hypoxia (∼3,750 m) (Hobbins et al., 2021). However, a notable distinction in the current study is that the overall perceived discomfort rating, perceived breathing difficulty and leg discomfort rating were all greater in high hypoxia compared to moderate hypoxia. Such findings are important when considering moderate hypoxia was able to significantly improve glucose regulation without inducing greater physiological and psychological distress. Furthermore, a significant decrease in self-reported pleasure that led to reduced exercise adherence was reported in people with overweight, despite exercising at greater intensity similar to normal-weight counterparts (Ekkekakis and Lind, 2006). Thus, from a perceptual standpoint, our results suggest that low intensity exercise combined with moderate hypoxia may be suitable for overweight adults to acquire metabolic health improvements without diminishing enjoyment and adherence due to greater perceptual discomfort. Further investigation regarding the manipulation of exercise intensity and duration of aerobic exercise under hypoxia on enjoyment and adherence in similar cohorts is required.

To elucidate possible mechanisms of how exercise and hypoxia can promote metabolic adaptations, we investigated the expression of the “exerkines,” apelin and FGF-21 in plasma. Apelin and FGF-21 are released with exercise-induced muscle contraction and are involved in metabolic regulation by controlling glucose and fat metabolism. As such, these exerkines are implicated in improving insulin sensitivity and preventing obesity and diabetes mellitus with exercise (Son et al., 2018; Khalafi et al., 2021). However, secretion of these exerkines after hypoxic exposure in combination with exercise remains poorly investigated. We found no significant changes in plasma apelin or FGF-21 post-exercise or between different conditions despite post-exercise glucose and insulin iAUC being significantly lower in moderate hypoxia. Previous studies have reported inconsistent findings in plasma apelin concentration following aerobic-based exercise under normoxic conditions in either healthy (Waller et al., 2019) or individuals with overweight or obesity (Son et al., 2019). Similarly, acute exercise under normoxic conditions has been shown to selectively increase plasma FGF-21 in lean individuals (Tanimura et al., 2016), but this response was attenuated in individuals with obesity (Slusher et al., 2015). Disparities in both plasma apelin and FGF-21 responses to acute exercise between studies may relate to differences in participant characteristics (i.e., age, physical activity levels and health status), exercise intensity and/or the timing of post-exercise sample collection and measurement (Slusher et al., 2015; Tanimura et al., 2016). Moreover, it cannot be ruled out that any significant changes in plasma apelin may have occurred outside our pre-, immediately post-, and 24 h post-exercise sampling window (Brame et al., 2015). Future studies with increasing post-exercise sampling frequency, as well as standardization and consistency in sample process time are needed to better clarify the patterns of circulating FGF-21 and apelin in response to exercise and hypoxia.

Several limitations of the present study are acknowledged. Firstly, our study recruited only overweight males with normal glycemia levels, therefore the results may not be able to be generalized to female and clinical cohorts. Furthermore, our study only included one type of exercise (aerobic cycle exercise) and one exercise intensity (low intensity). In this regard, it is plausible that alternative exercise modes, such as high-intensity interval training, or contractile types (i.e., walking, running), may induce different effects on blood glucose regulation to that currently observed with low-intensity aerobic exercise (Tee et al., 2023). Another limitation in the current study is that glucose and insulin responses were determined using OGTT rather than using the gold standard glucose tolerance assessment (hyperinsulinemic-euglycemic clamp technique) (DeFronzo et al., 1979), which was not possible for practical reasons. Lastly, previous studies have reported a positive effect of acute aerobic exercise on glucose tolerance in individuals with T2D and/or overweight (Mackenzie et al., 2011; Mackenzie et al., 2012a), and robust changes in blood glucose and exerkine regulation with exercise may be more apparent with repeated hypoxic exposure over the course of several weeks to months. While our study aimed to examine the acute effects of hypoxic stimuli combined with exercise, we acknowledge that repeated exercise sessions under hypoxic conditions may be required to observe more substantial changes in blood glucose responses.

## Conclusion

In conclusion, our results show that an acute bout of low-intensity exercise in combination with moderate hypoxia was most effective for improving post-exercise blood glucose regulation in healthy overweight adults. These results suggest that increasing hypoxic severity to 3,000 m and beyond may not be necessary to improve glucose regulation in such cohorts while also minimizing undue physiological stress and discomfort on participants. Thus, our findings provide an initial basis for determining safe and optimal hypoxic stimuli that can promote metabolic adaptations while reducing unnecessary physiological and psychological stress.

## Data Availability

The original contributions presented in the study are included in the article/Supplementary Materials, further inquiries can be directed to the corresponding author.

## References

[B1] Besse-Patin A. Montastier E. Vinel C. Castan-Laurell I. Louche K. Dray C. (2014). Effect of endurance training on skeletal muscle myokine expression in obese men: Identification of apelin as a novel myokine. Int. J. Obes. (Lond) 38 (5), 707–713. 10.1038/ijo.2013.158 23979219

[B2] Booth F. W. Roberts C. K. Laye M. J. (2012). Lack of exercise is a major cause of chronic diseases. Compr. Physiol. 2 (2), 1143–1211. 10.1002/cphy.c110025 23798298 PMC4241367

[B3] Brame A. L. Maguire J. J. Yang P. Dyson A. Torella R. Cheriyan J. (2015). Design, characterization, and first-in-human study of the vascular actions of a novel biased apelin receptor agonist. Hypertension 65 (4), 834–840. 10.1161/HYPERTENSIONAHA.114.05099 25712721 PMC4354462

[B4] Brydges C. R. (2019). Effect size guidelines, sample size calculations, and statistical power in gerontology. Innov. Aging 3 (4), igz036. 10.1093/geroni/igz036 31528719 PMC6736231

[B5] Burtscher J. Mallet R. T. Pialoux V. Millet G. P. Burtscher M. (2022). Adaptive responses to hypoxia and/or hyperoxia in humans. Antioxid. Redox Signal 37 (13-15), 887–912. 10.1089/ars.2021.0280 35102747

[B6] Callahan M. J. Parr E. B. Hawley J. A. Camera D. M. (2021). Can high-intensity interval training promote skeletal muscle anabolism? Sports Med. 51 (3), 405–421. 10.1007/s40279-020-01397-3 33512698

[B7] Carson B. P. (2017). The potential role of contraction-induced myokines in the regulation of metabolic function for the prevention and treatment of type 2 diabetes. Front. Endocrinol. (Lausanne) 8, 97. 10.3389/fendo.2017.00097 28512448 PMC5411437

[B8] Cengiz E. Tamborlane W. V. (2009). A tale of two compartments: Interstitial versus blood glucose monitoring. Diabetes Technol. Ther. 11, S11–S16. 10.1089/dia.2009.0002 19469670 PMC2903977

[B9] Chakravarty E. F. Hubert H. B. Lingala V. B. Fries J. F. (2008). Reduced disability and mortality among aging runners: A 21-year longitudinal study. Arch. Intern Med. 168 (15), 1638–1646. 10.1001/archinte.168.15.1638 18695077 PMC3175643

[B10] Chow L. S. Gerszten R. E. Taylor J. M. Pedersen B. K. van Praag H. Trappe S. (2022). Exerkines in health, resilience and disease. Nat. Rev. Endocrinol. 18 (5), 273–289. 10.1038/s41574-022-00641-2 35304603 PMC9554896

[B11] Coffey V. G. Hawley J. A. (2007). The molecular bases of training adaptation. Sports Med. 37 (9), 737–763. 10.2165/00007256-200737090-00001 17722947

[B12] Cohen J. (2013). Statistical power analysis for the behavioral sciences. UK: Routledge.

[B13] De Groote E. Britto F. A. Bullock L. Francois M. C D. E. B. Nielens H. (2018). Hypoxic training improves normoxic glucose tolerance in adolescents with obesity. Med. Sci. Sports Exerc 50 (11), 2200–2208. 10.1249/MSS.0000000000001694 29923910

[B14] DeFronzo R. A. Tobin J. D. Andres R. (1979). Glucose clamp technique: A method for quantifying insulin secretion and resistance. Am. J. Physiol. 237 (3), E214–E223. 10.1152/ajpendo.1979.237.3.E214 382871

[B15] Ekkekakis P. Lind E. (2006). Exercise does not feel the same when you are overweight: The impact of self-selected and imposed intensity on affect and exertion. Int. J. Obes. (Lond) 30 (4), 652–660. 10.1038/sj.ijo.0803052 16130028

[B16] Exercise, and Sports Science Australia (2019). Adult pre-exercise screening system (APSS). Available: https://www.essa.org.au/Public/ABOUT_ESSA/Pre-Exercise_Screening_Systems.aspx (Accessed October 13, 2021).

[B17] Farrell P. A. Wilmore J. H. Coyle E. F. Billing J. E. Costill D. L. (1979). Plasma lactate accumulation and distance running performance. Med. Sci. Sports 11 (4), 338–344. 10.1249/00005768-197901140-00005 530025

[B18] Fernandez Menendez A. Saudan G. Sperisen L. Hans D. Saubade M. Millet G. P. (2018). Effects of short-term normobaric hypoxic walking training on energetics and mechanics of gait in adults with obesity. Obes. (Silver Spring) 26 (5), 819–827. 10.1002/oby.22131 29575698

[B19] Garneau L. Aguer C. (2019). Role of myokines in the development of skeletal muscle insulin resistance and related metabolic defects in type 2 diabetes. Diabetes Metab. 45 (6), 505–516. 10.1016/j.diabet.2019.02.006 30844447

[B20] Geiger K. Muendlein A. Stark N. Saely C. H. Wabitsch M. Fraunberger P. (2011). Hypoxia induces apelin expression in human adipocytes. Horm. Metab. Res. 43 (6), 380–385. 10.1055/s-0031-1273767 21448846 PMC3108882

[B21] Gibala M. J. McGee S. L. (2008). Metabolic adaptations to short-term high-intensity interval training: A little pain for a lot of gain? Exerc Sport Sci. Rev. 36 (2), 58–63. 10.1097/JES.0b013e318168ec1f 18362686

[B22] Glassford A. J. Yue P. Sheikh A. Y. Chun H. J. Zarafshar S. Chan D. A. (2007). HIF-1 regulates hypoxia- and insulin-induced expression of apelin in adipocytes. Am. J. Physiol. Endocrinol. Metab. 293 (6), E1590–E1596. 10.1152/ajpendo.00490.2007 17878221 PMC2570255

[B23] Haufe S. Wiesner S. Engeli S. Luft F. C. Jordan J. (2008). Influences of normobaric hypoxia training on metabolic risk markers in human subjects. Med. Sci. Sports Exerc 40 (11), 1939–1944. 10.1249/MSS.0b013e31817f1988 18845972

[B24] Hawley J. A. Hargreaves M. Joyner M. J. Zierath J. R. (2014). Integrative biology of exercise. Cell 159 (4), 738–749. 10.1016/j.cell.2014.10.029 25417152

[B25] Hobbins L. Girard O. Gaoua N. Hunter S. (2021). Acute psycho-physiological responses to perceptually regulated hypoxic and normoxic interval walks in overweight-to-obese adults. J. Sci. Med. Sport 24 (5), 481–487. 10.1016/j.jsams.2020.11.011 33281095

[B26] Hoppeler H. Klossner S. Vogt M. (2008). Training in hypoxia and its effects on skeletal muscle tissue. Scand. J. Med. Sci. Sports 18, 38–49. 10.1111/j.1600-0838.2008.00831.x 18665951

[B27] Jung W. S. Kim S. W. Kim J. W. Park H. Y. (2021). Resistance training in hypoxia as a new therapeutic modality for sarcopenia-a narrative review. Life (Basel) 11 (2), 106. 10.3390/life11020106 33573198 PMC7912455

[B28] Khalafi M. Alamdari K. A. Symonds M. E. Nobari H. Carlos-Vivas J. (2021). Impact of acute exercise on immediate and following early post-exercise FGF-21 concentration in adults: Systematic review and meta-analysis. Horm. (Athens) 20 (1), 23–33. 10.1007/s42000-020-00245-3 33151509

[B29] Kim K. H. Kim S. H. Min Y. K. Yang H. M. Lee J. B. Lee M. S. (2013). Acute exercise induces FGF21 expression in mice and in healthy humans. PLoS One 8 (5), e63517. 10.1371/journal.pone.0063517 23667629 PMC3646740

[B30] Kon M. Nakagaki K. Ebi Y. Nishiyama T. Russell A. P. (2015). Hormonal and metabolic responses to repeated cycling sprints under different hypoxic conditions. Growth Horm. IGF Res. 25 (3), 121–126. 10.1016/j.ghir.2015.03.002 25900847

[B31] Krogh-Madsen R. Thyfault J. P. Broholm C. Mortensen O. H. Olsen R. H. Mounier R. (2010). A 2-wk reduction of ambulatory activity attenuates peripheral insulin sensitivity. J. Appl. Physiol. (1985) 108, 1034–1040. 10.1152/japplphysiol.00977.2009 20044474

[B32] Laurens C. Bergouignan A. Moro C. (2020). Exercise-released myokines in the control of energy metabolism. Front. Physiol. 11, 91. 10.3389/fphys.2020.00091 32116795 PMC7031345

[B33] Lei O. K. Sun S. Nie J. Shi Q. Kong Z. (2022). Sprint interval exercise improves cognitive performance unrelated to postprandial glucose fluctuations at different levels of normobaric hypoxia. J. Clin. Med. 11 (11), 3159. 10.3390/jcm11113159 35683546 PMC9181000

[B34] MacInnis M. J. Gibala M. J. (2017). Physiological adaptations to interval training and the role of exercise intensity. J. Physiol. 595 (9), 2915–2930. 10.1113/JP273196 27748956 PMC5407969

[B35] Mackenzie R. Elliott B. Maxwell N. Brickley G. Watt P. (2012a). The effect of hypoxia and work intensity on insulin resistance in type 2 diabetes. J. Clin. Endocrinol. Metab. 97 (1), 155–162. 10.1210/jc.2011-1843 21994967

[B36] Mackenzie R. Maxwell N. Castle P. Brickley G. Watt P. (2011). Acute hypoxia and exercise improve insulin sensitivity (S(I) (2*)) in individuals with type 2 diabetes. Diabetes Metab. Res. Rev. 27 (1), 94–101. 10.1002/dmrr.1156 21218513

[B37] Mackenzie R. Maxwell N. Castle P. Elliott B. Brickley G. Watt P. (2012b). Intermittent exercise with and without hypoxia improves insulin sensitivity in individuals with type 2 diabetes. J. Clin. Endocrinol. Metab. 97 (4), E546–E555. 10.1210/jc.2011-2829 22278428

[B38] Millet G. P. Debevec T. Brocherie F. Malatesta D. Girard O. (2016). Therapeutic use of exercising in hypoxia: Promises and limitations. Front. Physiol. 7, 224. 10.3389/fphys.2016.00224 27375500 PMC4902009

[B39] Potteiger J. A. Jacobsen D. J. Donnelly J. E. (2002). A comparison of methods for analyzing glucose and insulin areas under the curve following nine months of exercise in overweight adults. Int. J. Obes. 26 (1), 87–89. 10.1038/sj.ijo.0801839 11791151

[B40] Pramsohler S. Burtscher M. Faulhaber M. Gatterer H. Rausch L. Eliasson A. (2017). Endurance training in normobaric hypoxia imposes less physical stress for geriatric rehabilitation. Front. Physiol. 8, 514. 10.3389/fphys.2017.00514 28785224 PMC5517449

[B41] Rebrin K. Sheppard N. F. Jr. Steil G. M. (2010). Use of subcutaneous interstitial fluid glucose to estimate blood glucose: Revisiting delay and sensor offset. J. Diabetes Sci. Technol. 4 (5), 1087–1098. 10.1177/193229681000400507 20920428 PMC2956819

[B42] Ristow M. Schmeisser K. (2014). Mitohormesis: Promoting health and lifespan by increased levels of reactive oxygen species (ROS). Dose Response 12 (2), 288–341. 10.2203/dose-response.13-035 24910588 PMC4036400

[B43] Roach R. C. Hackett P. H. Oelz O. Bartsch P. Luks A. M. MacInnis M. J. (2018). The 2018 lake louise acute mountain sickness score. High. Alt. Med. Biol. 19 (1), 4–6. 10.1089/ham.2017.0164 29583031 PMC6191821

[B44] Saunders P. U. Telford R. D. Pyne D. B. Hahn A. G. Gore C. J. (2009). Improved running economy and increased hemoglobin mass in elite runners after extended moderate altitude exposure. J. Sci. Med. Sport 12 (1), 67–72. 10.1016/j.jsams.2007.08.014 18069063

[B45] Slusher A. L. Whitehurst M. Zoeller R. F. Mock J. T. Maharaj M. Huang C. J. (2015). Attenuated fibroblast growth factor 21 response to acute aerobic exercise in obese individuals. Nutr. Metab. Cardiovasc Dis. 25 (9), 839–845. 10.1016/j.numecd.2015.06.002 26141939

[B46] Son J. S. Chae S. A. Park B. I. Du M. Song W. (2019). Plasma apelin levels in overweight/obese adults following a single bout of exhaustive exercise: A preliminary cross-sectional study. Endocrinol. Diabetes Nutr. Engl. Ed. 66 (5), 278–290. 10.1016/j.endinu.2018.12.005 30827910

[B47] Son J. S. Chae S. A. Testroet E. D. Du M. Jun H. P. (2018). Exercise-induced myokines: A brief review of controversial issues of this decade. Expert Rev. Endocrinol. Metab. 13 (1), 51–58. 10.1080/17446651.2018.1416290 30063442

[B48] Taniguchi H. Tanisawa K. Sun X. Kubo T. Higuchi M. (2016). Endurance exercise reduces hepatic fat content and serum fibroblast growth factor 21 levels in elderly men. J. Clin. Endocrinol. Metab. 101 (1), 191–198. 10.1210/jc.2015-3308 26562755

[B49] Tanimura Y. Aoi W. Takanami Y. Kawai Y. Mizushima K. Naito Y. (2016). Acute exercise increases fibroblast growth factor 21 in metabolic organs and circulation. Physiol. Rep. 4 (12), e12828. 10.14814/phy2.12828 27335433 PMC4923231

[B50] Tee C. C. L. Chong M. C. Sundar V. Chok C. L. Md Razali M. R. Yeo W. K. (2022). Influence of exercise intensity and hypoxic exposure on physiological, perceptual and biomechanical responses to treadmill running. Eur. J. Sport Sci., 1–10. 10.1080/17461391.2022.2109066 35912915

[B51] Tee C. C. L. Cooke M. B. Chong M. C. Yeo W. K. Camera D. M. (2023). Mechanisms for combined hypoxic conditioning and divergent exercise modes to regulate inflammation, body composition, appetite, and blood glucose homeostasis in overweight and obese adults: A narrative review. Sports Med. 53 (2), 327–348. 10.1007/s40279-022-01782-0 36441492 PMC9877079

[B52] Waller J. D. McNeill E. H. Zhong F. Vervaecke L. S. Goldfarb A. H. (2019). Plasma apelin unchanged with acute exercise insulin sensitization. J. Sports Sci. Med. 18 (3), 537–543. 31427876 PMC6683609

[B53] Wiesner S. Haufe S. Engeli S. Mutschler H. Haas U. Luft F. C. (2010). Influences of normobaric hypoxia training on physical fitness and metabolic risk markers in overweight to obese subjects. Obes. (Silver Spring) 18 (1), 116–120. 10.1038/oby.2009.193 19543214

[B54] Wilber R. L. (2007). Application of altitude/hypoxic training by elite athletes. Med. Sci. Sports Exerc 39 (9), 1610–1624. 10.1249/mss.0b013e3180de49e6 17805095

[B55] World Health Organization (2021). Obesity and overweight. Available: https://www.who.int/en/news-room/fact-sheets/detail/obesity-and-overweight (Accessed October 10, 2022).

[B56] Wu G. Liu Y. Feng W. An X. Lin W. Tang C. (2020). Hypoxia-induced adipose lipolysis requires fibroblast growth factor 21. Front. Pharmacol. 11, 1279. 10.3389/fphar.2020.01279 32922298 PMC7456904

