# Supplementary figures and images for: Combined effects of exercise and different levels of acute hypoxic severity: A randomized crossover study on glucose regulation in adults with overweight
Source: Front Physiol. 2023 Apr 13;14:1174926. doi: 10.3389/fphys.2023.1174926 (PMC10133678; doi:10.3389/fphys.2023.1174926)

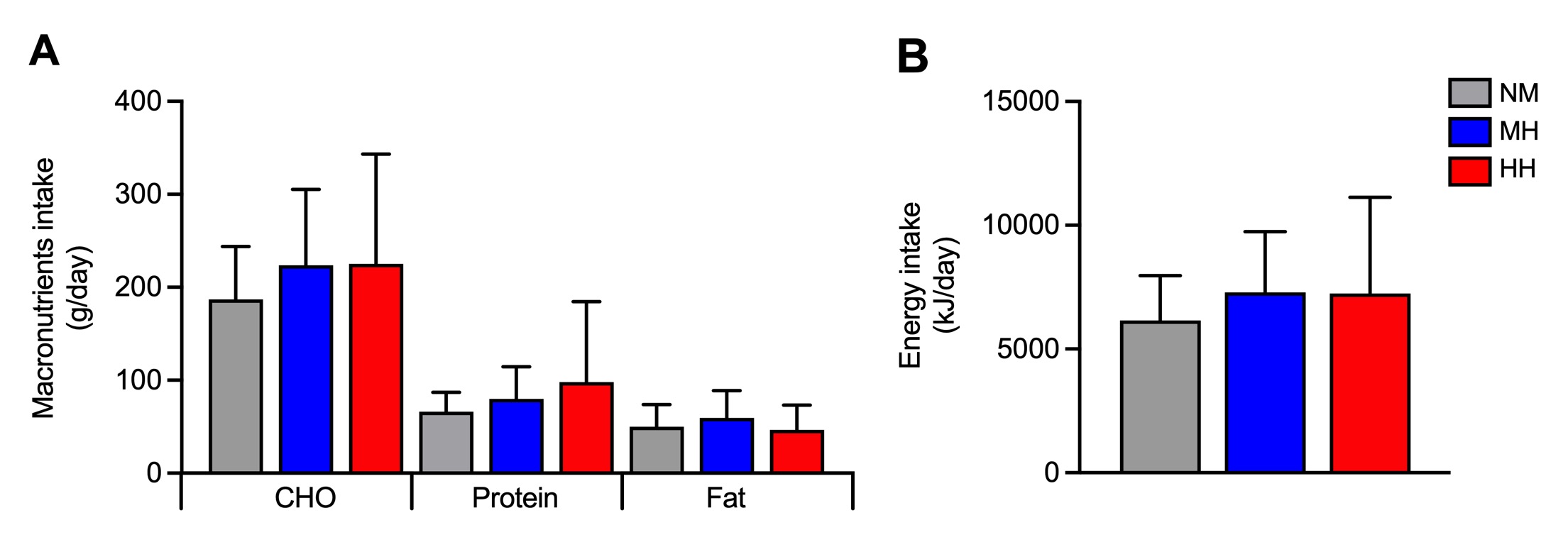

Supplement: Supplementary file 1 [file Image1.JPEG]
